# Impacted teeth: Their place is in the dental arch

**DOI:** 10.1590/2177-6709.24.6.020-026.oin

**Published:** 2019

**Authors:** Alberto Consolaro, Mauricio de Almeida Cardoso

**Affiliations:** 1 Universidade de São Paulo, Faculdade de Odontologia de Ribeirão Preto, Programa de Pós-Graduação em Odontopediatria (Ribeirão Preto/SP, Brazil).; 2 Universidade de São Paulo, Faculdade de Odontologia de Bauru (Bauru/SP, Brasil).; 3 Faculdade de Medicina e Odontologia São Leopoldo Mandic e do Programa de Pós-graduação em Ortodontia - Campinas, SP, Brazil.

**Keywords:** Pericoronal follicle, Unerupted teeth, Tooth eruption, Traction, Guided eruption

## Abstract

The starting point for the treatment of unerupted teeth should consider the fact that, biologically, the pericoronal follicle maintains the ability to release EGF and other mediators responsible for eruption over time. The eruptive events may be guided and directed, so that teeth may occupy the space prepared to receive them in the dental arch, as showed in the case presented to evidence the following principle to be considered in these cases: *“Regardless of the position of an unerupted tooth, it may be biologically directed to its place in the dental arch. The orthodontist should apply a mechanics to guide it and park it at its site.”*

## RESUMO

Os dentes não irrompidos devem ter como ponto de partida, para seu tratamento, o fato de que biologicamente o folículo pericoronário mantém ao longo do tempo a capacidade de liberar o EGF e outros mediadores responsáveis pela erupção. Pode-se guiar e direcionar os eventos eruptivos para que os dentes ocupem o seu espaço preparado para recebê-los na arcada dentária, como revela o caso apresentado para sedimentar o seguinte princípio a ser considerado para esses casos: *“Independentemente da posição que um dente não irrompido se apresente, há como, biologicamente, direcioná-lo para o seu local na arcada dentária. Cabe ao ortodontista aplicar uma mecânica que o leve até o local e lá o estacione."*

## PRINCIPLE APPLIED TO THE THERAPY OF UNERUPTED TEETH

Based on cell and tissue biology, as well as clinical and laboratory experience, it may be stated that: 

*“Regardless of the position of an unerupted tooth, it may be biologically directed to its site in the dental arch. The orthodontist should apply a mechanics to lead it and park it at its site.”*

Unerupted teeth present the tooth eruption organ around their crowns: the pericoronal follicle, even over the years! For several decades, it has been thought that the root was the fundamental structure for eruption, and even though this paper was published in 1980, many professionals are still unaware that the pericoronal follicle is the fundamental structure for tooth eruption.^1^

**Figure 1:**
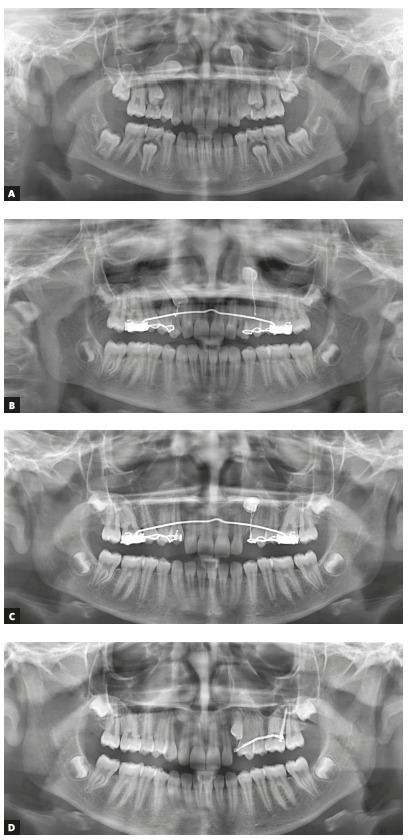
Unerupted upper canines due to abnormal positioning or ectopia. Observe the gradual inversion of positions by traction lever-arm mechanics. 10-year-old girl. A = at diagnosis, B = 6 months, C = 1 year and 1 month, and D = after 2 years and ten months.

**Figure 2:**
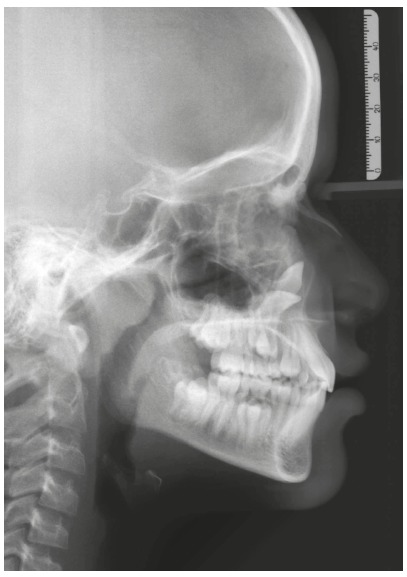
Cephalogram of the same case as the previous figure, evidencing the position of unerupted upper canines. The patient presents normal craniofacial growth pattern.

**Figure 3:**
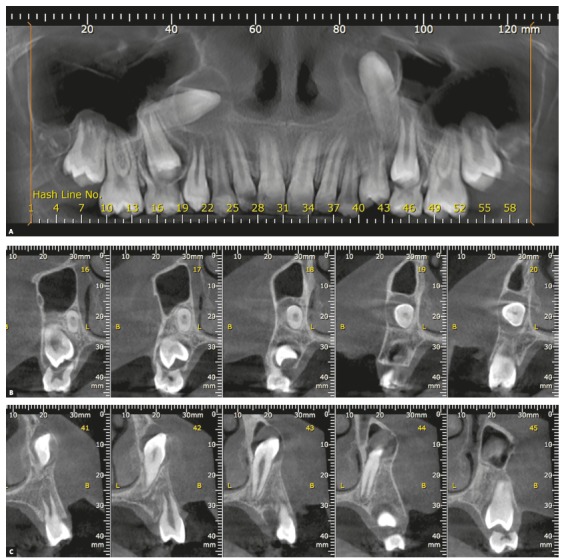
Longitudinal tomographic sections of upper canines presented in previous figures, highlighting the inverted positions and pericoronal spaces. A = panoramic reconstruction, B and C = sagittal sections of right and left upper canines, respectively.

There are no biological impairments (Figs 1, 2 and 3), since the pericoronal follicles retain their structural and functional conditions to secrete the mediators guided by the EGF, or Epidermal Growth Factor, responsible for the resorption of bone around it. When well directed mechanically, the teeth go through the bone according to the stimuli received.

Directing unerupted teeth to denser bone areas with lighter forces, the rate of tooth traction movement is similar to that usually observed in areas with normal density. This may happen even if there is a permanent or deciduous tooth, or even a root.

The opposite also applies. If unerupted teeth are directed with intense forces, the rate of tooth movement becomes very slow and even seems not to be moving! The compression of vessels reduces the quantity of blood to the follicular cells and tissues, and bone resorption becomes limited. 

Denser areas, and even teeth, do not retain teeth with mechanical stimuli if there is a pericoronal follicle with normal tissues. Ankylosed teeth after marked atrophy of the periodontal ligament do not move, even when mechanical stimuli are applied. The dentoalveolar ankylosis remains over time, naturally, with replacement tooth resorption that irreversibly attaches bone and teeth.

## PERICORONAL FOLLICLE: THE ERUPTION ORGAN MAY BE GUIDED

The soft tissue membrane that composes the pericoronal follicle is firmly attached to the crown surface by the reduced enamel epithelium and fills the radiolucent or hypodense space (Fig 3) around the crowns of unerupted teeth.^2^ This thin and delicate epithelium is supported and nourished by a loose to fibrous capsular connective tissue, and may even be hyalinized.

By comparison, in a simpler language, it may be stated that the reduced enamel epithelium and odontogenic epithelial islands of the follicle are two epithelial components that “moisten”, “wet” or increase the connective tissue level of EGF, the fundamental mediator of tooth eruption.

The most external part of pericoronal follicles present continuity with the adjacent bone. The pericoronal follicle tissues are inserted at the cementoenamel junction. At the cervical region, the epithelial ends with and over the enamel. The follicular connective portion, in turn, is inserted in the cementum and dentin gaps located right after the enamel margin.

In summary, the pericoronal follicle presents:

1. A tissue component represented by the reduced enamel epithelium adhered to enamel and cords and islands of odontogenic cells derived from the dental lamina.
2. A connective tissue that represents greater volume of follicular tissues, which after removal and outside the pericoronal space, is shaped as a membrane or a sac.
3. Continuous release of EGF by the epithelial component to stimulate the maintenance of its structure, while simultaneously maintaining the pericoronal space by stimulating bone resorption, keeping the bone distant from enamel.
4. Permeation of EGF that activates other mediator in cascade, making it fundamental for the mechanism of tooth eruption. The forces derived from the formation of teeth and growth stimulate greater secretion of EGF, which promotes directed bone resorption to guide the tooth in occlusal direction (Fig 1). An eruption guided by traction and applied mechanical forces may simulate these natural stimuli and redirect the tooth to its site in the dental arch.
5. Other functions as:
  a) “hiding” or protecting the enamel from resorption by clast cells;
  b) avoid the bone to form directly on the enamel surface;
  c) constitute the primary junctional epithelium by fusing with the mucosa and allowing the tooth to erupt in the oral cavity without exposure to the internal environment of the body, represented by the gingival connective tissue, to a highly contaminated environment.

## CRITERIA FOR EVALUATION OF A PERICORONAL SPACE IMAGE: IMAGE, THICKNESS, CONTOUR AND LIMIT

The image of the pericoronal space (Figs 1, 2 and 3) should:

- Be homogeneously radiolucent or hypodense, without radiopaque points or radiolucent demarcated areas, which may indicate evolution to odontogenic tumors;
- Present limit with the adjacent bone represented by a uniform and continuous radiopaque line. If this line is interrupted and/or with scalloped-like images, it may represent evolution to odontogenic cysts and tumors;
- Present uniform thickness of the pericoronal space contour and symmetrical shape in relation to the tooth crown. The formation of thicker areas, with ruffled shapes, may evidence evolution to odontogenic cysts and tumors;
- Present thickness ranging from smaller than 1 mm up to 5.6 mm.^2^ ^,^ ^3^ Measurements different than these may indicate dentigerous cyst or other follicular disorder.

In the evaluation of the pericoronal space image, it should be emphasized that pericoronal follicle disorders may be very small and be present even when the pericoronal space presents apparently normal thickness. 

## A CASE REPORT FOR CLINICAL DEMONSTRATION

**Figure 4:**
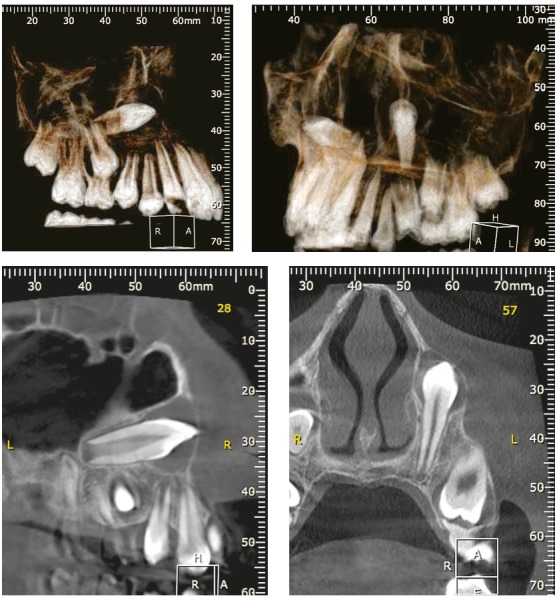
3D reconstructions and coronal tomographic sections of upper canines presented in previous figures, highlighting the inverted positions and pericoronal spaces.

**Figure 5:**
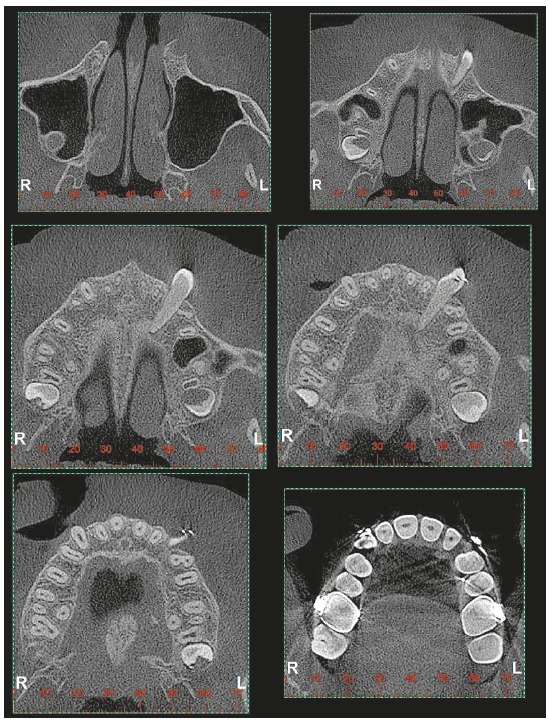
Axial tomographic sections of upper left canine, crossing the alveolar process in horizontal position with the crown on the buccal side, after mechanics for 1 year and 10 months. Clinical case of previous figures.

**Figure 6:**
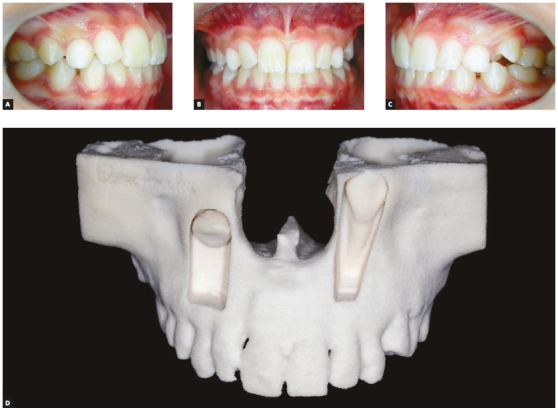
Clinical aspect of patient of previous figures at A, B and C upon diagnosis, and at D prototyping accurately demonstrating the spatial positioning of unerupted upper canines.

Figures 1 to 6 present a case in which two canines are buccally positioned with their crowns turned toward the frontal region. Notwithstanding, the adequate planning of guided eruption allowed adequate repositioning to reach its site in the dental arch by application of a traction lever-arm mechanics. 

Previous studies^2^ ^,^ ^3^ revealed that, regardless of the patient age, the follicular structures maintain their function and epithelial component represented by the reduced enamel epithelium and odontogenic epithelial islands derived from the dental lamina. In some teeth, the epithelium presents metaplasia to stratified pavement and achieves new cell layers.^1^

The “insight” of this paper is to induce a review of cases to reach the following starting point for therapeutic decisions involving unerupted teeth that should be filling their space in the dental arch:

*“Regardless of the position of an unerupted tooth, it may be biologically directed to its site in the dental arch. The orthodontist should apply a mechanics to lead it and park it at its site.”*

## FINAL CONSIDERATIONS

Every unerupted tooth, at any time and provided its pericoronal follicle and periodontal tissues are healthy, may be submitted to guided eruption for traction into its space in the dental arch. Orthodontics has enough technology and knowledge for that purpose!
